# Bioactive fraction from *Plumeria obtusa* L. attenuates LPS-induced acute lung injury in mice and inflammation in RAW 264.7 macrophages: LC/QToF-MS and molecular docking

**DOI:** 10.1007/s10787-023-01144-w

**Published:** 2023-02-11

**Authors:** Yousra T. Eloutify, Riham A. El-Shiekh, Khaled Meselhy Ibrahim, Ahmed R. Hamed, Ahmed A. Al-Karmalawy, Aya A. Shokry, Yasmine H. Ahmed, Bharathi Avula, Kumar Katragunta, Ikhlas A. Khan, Meselhy R. Meselhy

**Affiliations:** 1 Department of Pharmacognosy, Faculty of Pharmacy, Cairo University, Kasr el Aini St., Cairo, 11562 Egypt; 2 Chemistry of Medicinal Plants Department and Biology Unit, Central Lab for the Pharmaceutical and Drug Industries Research Institute, National Research Centre, 33 El-Bohouth St, Giza, 12622 Dokki Egypt; 3 Pharmaceutical Chemistry Department, Faculty of Pharmacy, Ahram Canadian University, 6th of October City, Giza, 12566 Egypt; 4 Department of Pharmacology, Faculty of Veterinary Medicine, Cairo University, Cairo, Egypt; 5 Cytology and Histology Department, Faculty of Veterinary Medicine, Cairo University, Giza, Egypt; 6 National Center for Natural Products Research, School of Pharmacy, University of Mississippi, University, MS 38677 USA; 7 Division of Pharmacognosy, Department of BioMolecular Sciences, School of Pharmacy, University of Mississippi, University, MS 38677 USA

**Keywords:** *Plumeria obtusa* L., RAW 264.7 macrophages, LPS-induced ALI, Immunohistochemistry, LC/QToF, Molecular docking

## Abstract

In this study, the anti-inflammatory effects of the methanolic extract (TE) of *Plumeria obtusa* L. (aerial parts) and its fractions were evaluated in vitro, and active fraction was evaluated in vivo. Among tested extracts, dichloromethane fraction (DCM-F) exhibited the strongest inhibition of lipopolysaccharide (LPS)-induced nitric oxide (NO) in RAW 264.7 macrophages. The effect of DCM-F on LPS-induced acute lung injury (ALI) in mice was studied. The animals were divided into five groups (*n* = 7) randomly; Gp I: negative control, GP II: positive control (LPS group), GP III: standard (dexamethasone, 2 mg/kg b.wt), GP IV and V: DCM-F (100 mg/kg), and DEM-F (200 mg/kg), respectively. DCM-F at a dose of 200 mg/kg suppressed the ability of LPS to increase the levels of nitric oxide synthase (iNOS), NO, tumor necrosis factor-α (TNF-α), and interleukin 6 (IL-6), as measured by ELISA. In addition, the expression of cyclooxygenase-2 (COX-2) was reduced (determined by immunohistochemistry) and the level of malondialdehyde (MDA) was decreased while that of catalase was restored to the normal values. Furthermore, the histopathological scores of inflammation induced by LPS were reduced. Twenty-two compounds were tentatively identified in DCM-F using LC/ESI-QToF with iridoids, phenolic derivatives and flavonoids as major constituents. Identified compounds were subjected to two different molecular docking processes against iNOS and prostaglandin E synthase-1 target receptors. Notably, protoplumericin A and 13-*O*-coumaroyl plumeride were the most promising members compared to the co-crystallized inhibitor in each case. These findings suggested that DCM-F attenuates the LPS-induced ALI in experimental animals through its anti-inflammatory and antioxidant potential.

## Introduction

Acute lung injury (ALI) is an inflammatory disease of the lung characterized by the destruction of the alveolar endothelial and epithelial barriers, neutrophilic infiltration at pulmonary sites and non-cardiogenic edema. Globally, ALI is a life-threatening disease that imposes a substantial health burden in the intensive care units and is the main cause of acute respiratory distress (ARD) syndrome. Although progress has been made in treatments, the morbidity and mortality rates of ALI sill very high (30–40%) (Ali et al. 2020). Currently, treatment is primarily focused on treatment of the underlying condition and bedside care, including mechanical ventilation and corticosteroid administration. The pharmacological strategies have not been effective in reducing mortality (Laffey and Kavanagh 2000; Meng et al. 2022; Bo et al. 2021). Accordingly, new therapies with high safety and efficacy are needed to enhance the clinical outcomes of ALI patients (Wang et al. 2020).

Lipopolysaccharide (LPS), an endotoxin secreted in bacteria, is used as a potent inducer of inflammation. The interaction of LPS and the lung is very important; it stimulates the secretion of numerous inflammatory mediators by triggering the neutrophils and macrophages (Fujihara et al. 2003). LPS-induced inflammation has been widely used in the assessment of the anti-inflammatory effects of agents in both in vitro and in vivo models (Kim et al. 2015; Shokry et al. 2022; Nworu et al. 2012).

*Plumeria obtusa* L. (family Apocynaceae) is a large shrub or small tree with attractive and fragrant flowers and producing a milky sap. The plant is widely cultivated in tropical areas in Asia, Hawaii, and Eastern Africa (Shinde et al. 2014; Bihani and Mhaske 2020). The plant organs were reported to have many pharmacological activities such as antibacterial, anti-inflammatory, antiproliferative, cytotoxic, antiulcerogenic, antipyretic, antioxidant, and wound healing activities (Bihani 2021). In traditional medicine, the plant is used as a remedy for wounds, ulcers, leprosy, herpes zoster, tinea, toothache, cracked heels, boils, sores, herpetic lesions, syphilis and skin diseases. Bark and latex are used as diuretic and purgative (Lim 2012; Wong et al. 2011; Krishen 2006; Nuraeni and Rustaman 2019).

Of the major constituents previously identified and isolated from *P. obtusa* are iridoids such as plumericin, plumieride and derivatives, flavonoids such as rutin, kaempferol and quercetin, and phenolic acids such as gallic, protocatechuic, chlorogenic, and sinapic acids. Also, triterpenoids such as betulinic acid, oleanolic acid, and *α*-amyrin are very common chemical constituents of the *Plumeria* species (Bihani 2021).

The anti-inflammatory activity of the total ethanolic extract of *P. obtusa* L. stem bark was studied on carrageenan-induced paw edema and cotton pellet granuloma pouch model in rats (at 100, 200, and 400 mg/kg) (Lotankar et al. 2016). The authors reported that doses of 400 mg/kg demonstrated better and comparable results to that of standard indomethacin (10 mg/kg). The present study is to investigate the anti-inflammatory activity of *P. obtusa* L. (aerial parts) on LPS-induced ALI in mice through a bioassay-guided fractionation and to identify the active fraction using LC/MS and molecular docking.

## Materials and Methods

### Plant material

*Plumeria obtusa* L., aerial parts were collected from Mazhar botanical garden, Giza, Egypt in October 2020. The plant material was kindly identified by Ms. Therese Labib, a Botanical Specialist and Consultant at Orman botanic garden. A voucher specimen (No 3-07-2022) was deposited at the herbarium of Pharmacognosy, Faculty of Pharmacy, Cairo University, Cairo, Egypt. The aerial parts were washed with tap water to remove debris, dust, and solid materials, and left to dry in shade (14 days, ≈ 25 °C). The dried plant material was powdered, sieved to 80 mesh, and the powder was stored in a sealed container till use.

### Solvents

Solvents used for extraction and preparation of the bioactive fraction were of analytical grade and supplied by Piochem Company (Cairo, Egypt). The selected solvents vary between methanol, dichloromethane, ethyl acetate, acetonitrile, formic acid, and glacial acetic acid.

### Extraction and fractionation

The powdered plant material (3.0 kg) was extracted with MeOH (3 × 7 L) using Ultra Turrax T50 homogenizer (Germany) at room temperature for 72 h. The methanolic extract was filtered and evaporated under reduced pressure to give 300 g of dry residue (TE). A part of the residue (100 g) was suspended in water (250 mL) and fractionated between dichloromethane (3 × 750 mL). The dichloromethane layer was evaporated under reduced pressure to yield a dichloromethane fraction (DCM-F, 56 g). The aqueous layer was applied on the top of a Diaion HP-20 column (80 g). Elution was started with distilled water (1 L), 50% methanol/water (1.5 L), and 100% methanol (1.5 L), and fractions were evaporated under reduced pressure to yield water fraction (W-F, 17 g), 50% methanol/water fraction (MW-F, 13 g), and methanol fraction (M-F, 12 g).

#### Liquid chromatography diode array detector-quadrupole time-of-flight mass spectrometry (LC-DAD-QToF) analysis of DCM-F

### Sample preparation

Part of DCM-F residue was prepared in HPLC-grade methanol, filtered, and placed into LC vials prior to analysis. Acetonitrile, methanol, formic acid used are of HPLC grade, and water was purified using a Milli-Q system (Millipore, Bedford, MA, USA).

### Instrumentation

The liquid chromatographic system used for analysis was an Agilent Series 1290. The separation of the compounds in DCM-F was carried out on an Acquity UPLC^™^ HSS C18 column (100 mm × 2.1 mm I.D., 1.8 µm). The mobile phase was composed of water (A) and acetonitrile (B), both containing 0.1% formic acid. The flow rate was 0.23 mL/min. Analysis was performed using the gradient elution of 90%A/10%B to 65%A/35%B in 20 min, in next 5 min to 100%B. Each run was followed by a 3 min wash with acetonitrile and followed by equilibration period of 5 min with 90%A/10%B. Two microliters of sample were injected, and the temperature of column was maintained at 40 °C.

The LC system was coupled to a QToF-MS-MS (Model #G6545B, Agilent Technologies, Santa Clara, CA, USA) fitted with an Electrospray Ionization Source (ESI) enhanced with a Jet Stream Technology Ion Source (AJS) using nitrogen as the drying gas at a flow of 13 L/min and 300 °C. Nebulizer pressure was maintained at 30 psig with sheath gas at 12 L/min and 400 °C. Source voltages were set at: capillary voltage, 4000 V; nozzle, 0 V; skimmer 65 V; Oct RF 750 V; and fragmentor 150 V. Agilent MassHunter Acquisition Software Ver. A.10.1 and Mass Hunter Qualitative Analysis Software Ver. B.10.00 were used to control instrument operation, data acquisition, analysis and processing of data. Both positive and negative ion mass spectra as well as all-ion mass spectra were obtained over the range of m/z 50 to 1700. All-ion MS data was obtained at a collision energy of 0 eV for experiment 1 and at 45 eV for experiment 2. Measured accurate masses were determined using references for positive ion MS of protonated purine (m/z = 121.0509) and HP-921; protonated hexakis (1H, 1H, 3H-tetrafluoropropoxy) phosphazine (m/z = 922.0098). For negative ion mass references were deprotonated trifluoroacetic acid-TFA (m/z = 112.9856) and TFA adducted HP-921 (m/z = 1033.9881) (Avula et al. 2014).

### Chemicals and reagents

Lipopolysaccharides (LPS’s) of *E. coli* and dexamethasone sod. phosphate reagents were purchased from Sigma Aldrich (St. Louis, MO, USA). Mouse IL- 6 (Cat# SEA079Ra), and TNF-α (Cat# abx050220) ELISA kits were obtained from Sinogeneclon Co. Ltd. (Hangzhou, China). Malondialdehyde (MDA) (Cat# K739-100), catalase (Cat# MBS8243260), nitric oxide (NO) (Cat# K252-200), iNOS (Cat# MBS723617) kits were from Bio-diagnostic Company, Dokki, Egypt.

### Cell culture

Murine macrophages RAW264.7 cells (ATCC^®^) were maintained in complete Dulbecco’s Modified Eagle’s Medium (DMEM, Corning, USA) supplemented with 10% fetal bovine serum, penicillin (100 U/mL), streptomycin sulphate (100 µg/mL) and 2 mM L-glutamine in a humidified 5% CO_2_ incubator. For passaging and treatment, cells were washed with phosphate buffered saline and scrapped off the flasks using sterile scrappers (SPL, Spain).

### In vitro anti-inflammatory assay on RAW 264.7 macrophages

RAW 264.7 cell stock (0.5 × 10^6^ cells/mL) were seeded into 96-well microwell plates and incubated for overnight. At the next day, non-induced triplicate wells received medium with the sample vehicle (DMSO, 0.1%, by volume). Inflammation group of triplicate wells received the inducer of inflammation [lipopolysaccharide (LPS) as 100 ng/mL in complete culture media]. Preliminary screening was performed on total extract and fractions using a single concentration of samples (100 µg/ml) dissolved in DMSO and diluted into culture media containing LPS (Final concentration of DMSO = 0.1%v/v. Indomethacin (INDO, 0.25 mM) was used as an anti-inflammatory positive control. After 24 h of incubation, Griess assay (Yoo et al. 2012) was used to determine nitric oxide (NO) in all wells. Equal volumes of culture supernatants and Griess reagent were mixed and incubated at room temperature for 10 min to form the colored diazonium salt and read at absorbance of 520 nm on a Tristar2 lb^™^ microplate reader (Berthold, Germany). NO inhibition % of test extract was calculated relative to the LPS-induced inflammation group (% NO inhibition of LPS^+^ = zero).

### ***IC***_***50***_*** determination***

DCM-F stock solution was diluted into culture media as described above and pipetted onto triplicate wells to receive increasing concentrations (6.25, 12.5, 25.0, 50.0 and100.0 µg/mL) of the fraction with final concentration of DMSO being 0.1% v/v. GraphPad Prism V6 (San Diego, USA) was used to calculate the 50% inhibitory concentration of DCM-F using non-linear regression curve fit.

### Western blotting analysis of iNOS protein expression

RAW 264.7 cells were cultured and treated as previously described (Hamed et al. 2021) with minor modifications. Briefly, cells were cultured overnight on 6-well plates as 1.5 × 10^6^ cells/well. Cells were then treated with increasing concentrations (25, 50 and 100 µg/ml) of DCM-F dissolved in DMSO and diluted into culture medium containing 100 ng/ml of LPS with negative and positive controls as mentioned above. Following 24 h exposure time, cells were washed briefly with ice cold phosphate buffered saline (PBS, 1 ml/well). PBS was then discarded and 120 µls of lysis buffer (RIPA buffer containing 1 × dilution of Halt^®^ phosphatase and protease inhibitor cocktail) were added to each well. Cells were then scrapped on ice with scrapper, collected and incubated on ice in microcentrifuge tubes for 20 min with occasional brief vortexing. Cell lysates were briefly sonicated on ice (20% amplitude for 10 s). Sonicates were centrifuged (15,000 × g for 10 min at 4 °C). Supernatants were collected and estimated for total protein contents using BCA assay kit (Thermofisher Scientific, USA). Proteins in the cell lysates (30 µg) were separated on 10% SDS-PAGE gel (Bio-Rad Mini-PROTEAN Tetra cell, USA) and then transferred onto nitrocellulose membrane using a Trans-blot mini module (Bio-Rad, USA). The nitrocellulose membrane was blocked by using 5% skim milk for 1 h at room temperature, followed by an overnight incubation at 4 °C with either iNOS primary antibody (1:1000, Biomatik, Canada) or β-actin (1:2000, Thermofisher Scientific, USA). Following three × 5 min washes using Tris buffer saline Tween 20 (TBST), the membranes were incubated with 1:10,000 dilution of horseradish anti-rabbit or anti-mouse peroxidase-conjugated secondary antibodies for 1 h at room temperature. After three TBST washes, membrane proteins were revealed using the Enzyme Chemiluminescence (ECL) western blotting detection substrate. Protein bands were imaged using UVP Bio spectrum imaging system (Analytik Jena, UK).

#### In vivo* acute lung inflammation*

### Animals

Male mice (Age: 6–8 weeks, weight: 20–25 g) were obtained from the National Research Center, Egypt. Mice were given standard feed and water. The animals were housed in a sterilized animal house with specific temperature (24 ± 2 °C) and relative humidity (45–55%). All the animals were kept in these conditions 5 days before beginning the experiment.

### The experimental design

The protective effects of DCM-F of *Plumeria obtusa* L. were investigated according to previous references (Alagan et al. 2019; Shokry et al. 2022). Where, 35 mice were divided into five groups randomly (each of 7 mice): negative control, positive control (LPS group), standard (dexamethasone, DEXA, 2 mg/kg b.wt), DCM-F (at a dose of 100 mg/kg b.wt and 200 mg/kg b.wt). This experiment was carried out according to the instructions of the National Institutes of Health Guide for Care and Use of Laboratory Animals office (IACUC No.: Vet CU 2009 2,022,474).

During the first 7 days of the experiment, all mice in the first and second group were administrated orally with normal saline, those in the third group were given DEXA, while those in the fourth and fifth group were given DCM-F (100 mg/kg and 200 mg/kg, respectively) (Lotankar et al. 2016). Model mice of acute lung inflammation (ALI) was prepared by intranasal administration of 10 μL of LPS (4 mg/mL solution) after 30 min of oral administration of the drugs using oral gavage on day 7 of the experiment (Shokry et al. 2022). The negative control group was challenged with phosphate-buffered saline intranasally. After 6 h, the mice were anaesthetized using thiopental sodium (40 mg/kg, i.p.), then euthanized by cervical dislocation (Al-Massri et al. 2018), and lungs were collected. The homogenate was prepared from the lungs of mice for the assay of iNOS, NO, MDA, and catalase activity. In addition, the levels of TNF-α and IL-6 were measured using different rat ELISA kits according to the manufacturer's instructions.

### Histopathological study

#### Light microscopy

After the euthanasia of mice, the right and left lungs were quickly removed, inflated, and then fixed with 10% neutral buffered formalin. Fixed samples were dehydrated, followed by xylene, and embedded in paraffin. Sections (3–4 μm thick) were prepared, deparaffinized and stained with hematoxylin and eosin (H&E) for histopathological examination (Bancroft and Gamble 2013) and immunohistochemistry. Evaluations of lung injury and inflammatory cell infiltration were conducted using the modified scoring system according to (Tianzhu et al. 2014).

#### Immunohistochemical examination

Cyclooxygenase 2 protein (COX-2) was used as a marker for inflammation and immunohistochemical examination was performed as reported by (Côté et al. 1993).

#### Image analysis to evaluate immunohistochemical observations (area percentage)

Sections stained with anti-COX-2 were analyzed using a digital Leica Quin 500Â image analysis system (Leica Microsystems, Switzerland) housed at the Faculty of Dentistry, Cairo University. The image analyzer was automatically calibrated to convert pixels into units of area (μm^2^). COX-2 immunostaining was presented as a percentage of the total area in a standard measuring frame over ten independent fields from different slides in each group at 400 × magnification. All areas with positive immunohistochemical staining were evaluated, regardless of the intensity. The mean values and standard error (SE) obtained for each specimen were statistically analyzed.

### Docking studies

Two different molecular docking studies were performed to investigate the affinities of the 26 identified compounds from DCM-F toward both iNOS and prostaglandin E synthase-1 target receptors. Besides, the co-crystallized inhibitor of each target receptor was inserted as a reference standard in the corresponding docking process.

At the start, the identified compounds were sketched using the ChemDraw Professional 17.0 and prepared using the MOE (Inc 2016; Abdallah et al. 2021). 3D hydrogenation and energy minimization steps were carried out as previously reported (Ma et al. 2021). The prepared compounds were inserted into two databases together with the co-crystallized inhibitor of each target receptor in each case.

At the same time, both target protein receptors (iNOS and prostaglandin E synthase-1) were extracted and downloaded from the Protein Data Bank (IDs; 1M9T (Rosenfeld et al. 2002) and 5K0I (Kuklish et al. 2016), respectively). Each protein was opened in the MOE window and prepared for docking through correction, 3D hydrogenation, and energy minimization as described before (El Gizawy et al. 2021).

Finally, each previously prepared database was uploaded in the corresponding docking process applied as a general one and keeping the program specifications as illustrated earlier (Khattab and Al-Karmalawy 2021). Moreover, the docking program validation was confirmed by redocking the co-crystallized inhibitor of each target protein in its binding pocket. Both low values of root mean square deviation (RMSD) < 2, and closely similar binding modes compared to the co-crystallized inhibitor in each case were obtained as well (Al-Karmalawy et al. 2021; Srour et al. 2023; Fawazy et al. 2022).

### Statistical analysis

All quantitative results were analyzed using the SPSS version 17.0 for Windows. Data were presented as mean ± SD. Comparisons among multiple group means were performed using a one-way analysis of variance, followed by a Duncan's multiple comparison test. Statistical significance was set at *P* ≤ 0.05.

## Results

### LC/MS identification of the active fraction DCM-F

**Fig. 1:**
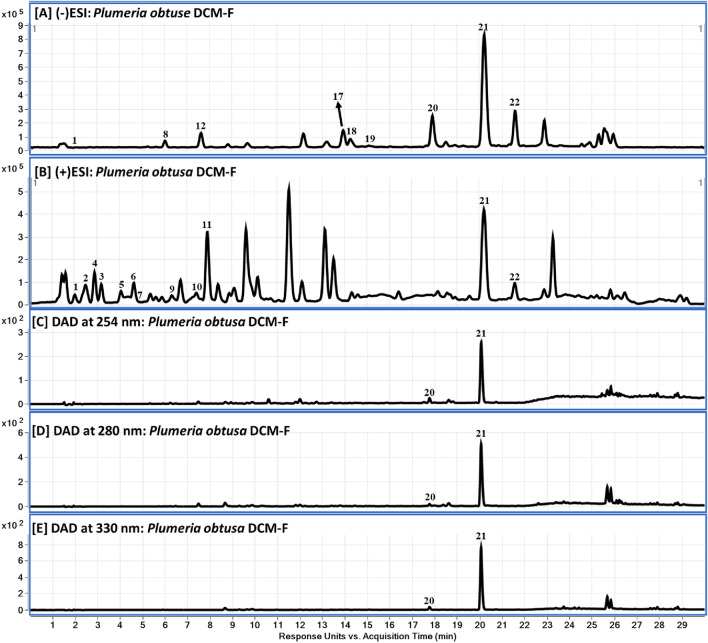
LC-DAD-QToF chromatograms of DCM-F of *Plumeria obtuse* L. (aerial parts): **A**, **B** QToF-MS base peak chromatograms in negative and positive modes, and **C**–**E** DAD chromatograms at 254, 280 and 330 nm, respectively

The chemical profile of the DCM-F was clarified using LC-DAD-QToF and MS/MS (Fig. 1 and Table 1). Twenty-two compounds were identified in the active fraction (DCM-F) including 9 iridoids, 7 phenolic acids, 2 flavonoids as the major constituents.

**Table 1:** Tentative identification and characterization of phytochemical compounds in DCM-F of *Plumeria obtusa* L. (aerial parts) using LC-QToF (in positive and negative ionization modes)

#	RT (min)	Compound name	Mol. formula	Exact mass [M]	[M + H]^+^	Fragment ions	[M−H]^−^	Fragment ions
1	2.00	Quinaldic acid	C_10_H_7_NO_2_	173.0477	174.0554 (174.0550)	143.0342 [M + H–CH_3_O]^+^	172.0404 (172.0404)*	128.0488 [M−H–CO_2_]^−^
2	2.40	Plumerianine/Plumericidine	C_13_H_13_NO_3_	231.0895	232.0967 (232.0968)	186.0903 [M + H–CH_2_O_2_]^+^, 170.0593 [M + H–CH_2_O_2_–CH_4_]^+^, 142.0643 [M + H–CH_2_O_2_–CH_4_–CO]^+^, 130.0645 [M + H–CH_2_O_2_–CH_4_–C–CO]^+^	–	–
3	3.05							
4	2.64	Phenylalanine	C_9_H_11_NO_2_	165.0790	166.0858 (166.0863)	120.0477 [M + H–C_2_H_6_O]^+^	164.0717 (164.0717)	–
5	4.14	Hydroxyquinoline	C_9_H_7_NO	145.0528	146.0605 (146.0600)	–	144.0457 (144.0455)	–
6	4.73	Chlorogenic acid/Neochlorogenic acid	C_16_H_18_O_9_	354.0951	355.1031 (355.1024)	163.0390 [M + H–C_7_H_12_O_6_]^+^	353.0881	191.0564 (quinic acid)
7	4.73	Quinic acid	C_7_H_12_O_6_	192.0634	–	–	191.0561	–
8	6.00	Dihydroxycoumarin	C_9_H_6_O_4_	178.0266	179.0328 (179.0339)	–	177.0196 (177.0193)	149.0243 [M−H–CO]^−^, 133.0297 [M−H–CO_2_]^−^, 121.0295 [M−H–2CO]^−^, 105.0348 [M−H–CO_2_–CO]^−^,
9	6.60	Coumaroylquinic acid	C_16_H_18_O_8_	338.1002	339.1074 (339.1074)	193.0491 [M + H–C_6_H_10_O_4_]^+^, 165.0541 [M + H–C_6_H_10_O_4_–CO]^+^, 147.0437 [M + H–C_6_H_10_O_4_–CO–H_2_O]^+^	337.0945 (337.0929)	191.0556 [M−H–C_6_H_10_O_4_]-
10	7.48	Plumericin/Isoplumericin	C_15_H_14_O_6_	290.0790	291.0869 (291.0863)	263.0913 [M + H–CO]^+^, 245.0806 [M + H–CO–H_2_O]^+^, 231.0650 [M + H–CO–H_2_O–CH_2_]^+^, 213.0545 [M + H–CO–2H_2_O–CH_2_]^+^, 203.0608 [M + H–2CO–H_2_O–CH_2_]^+^	289.0725 (289.0718)	–
11	7.61							
12	7.55	4-*O*-(3'-*O*-α-D-Glucopyranosyl)-caffeoyl quinic acid	C_22_H_28_O_14_	516.1479	–	–	515.1410 (515.1406)	191.0564
13	9.30	Quercetin rutinoside	C_27_H_30_O_16_	610.1534	611.1611 (611.1609)	465.1026 [M + H–C_6_H_10_O_4_]^+^, 303.0493 [M + H–C_6_H_10_O_4_–C_6_H_10_O_5_]^+^	609.1489 (609.1461)	300.0291 [M−H–C_12_H_21_O_9_]^−^
14	9.85	Plumenoside	C_20_H_24_O_11_	440.1319	–	–	439.1243 (439.1246)	259.0609 [M−H–C_6_H_12_O_6_]^−^, 215.0714 [M−H–C_6_H_12_O_6_–CO_2_]^−^,
15	11.0	Kaempferol rutinoside	C_27_H_30_O_15_	594.1585	595.1659 (595.1657)	287.0550	593.1517 (593.1512)	284.0330, 285.0393
16	12.91	Dicaffeoylquinic acids	C_25_H_24_O_12_	516.1268	–	–	515.1192 (515.1195)	353.0883 [M−H–C_9_H_6_O_3_]^−^, 191.0561[M−H–2C_9_H_6_O_3_]^−^, 173.0460 [M−H–2C_9_H_6_O_3_–H_2_O]^−^
17	14.0	Hydroxybenzoic acid	C_7_H_6_O_3_	138.0317	–	–	137.0145 (137.0244)	93.0291 [M−H–CO_2_]^−^
18	14.6	Protoplumericin A	C_36_H_42_O_19_	778.2320	796.2664 (796.2658) [M + NH_4_]^+^	361.1280 [M + H–C_17_H_22_O_12_]^+^, 147.0440 [M + H–C_17_H_22_O_12_–C_10_H_14_O_5_]^+^	777.2245 (777.2248)	615.1715 [M−H–C_6_H_10_O_5_]^−^, 343.1399 [M−H–C_6_H_10_O_5_–C_14_H_8_O_6_]^−^, 163.0401 [M−H–C_6_H_10_O_5_–C_14_H_8_O_6_–C_7_H_16_O_5_]^−^
19	15.15							
20	17.90	13-*O*-Caffeoylplumieride	C_30_H_32_O_15_	632.1741	633.1816 (633.1814)	471.1287 [M + H–C_6_H_10_O_5_]^+^, 325.0923 [M + H–C_6_H_10_O_5_–C_9_H_6_O_2_]^+^, 291.0864 [M + H–C_6_H_10_O_5_–C_9_H_6_O_2_–H_2_O]^+^, 231.0655 [M + H–C_6_H_10_O_5_–C_9_H_6_O_2_–H_2_O–C_2_H_4_O_2_]^+^, 163.0388 [M + H–C_6_H_10_O_5_–C_9_H_6_O_2_–H_2_O–C_2_H_4_O_2_–C_4_H_4_O]^+^	631.1686 (631.1688)	469.1142 [M−H–C_6_H_10_O_5_]^−^, 289.0714 [M−H–C_6_H_10_O_5_–C_9_H_6_O_2_–H_2_O]^−^, 213.0548 [M−H–C_6_H_10_O_5_–C_9_H_6_O_2_–H_2_O–C_2_H_4_O_3_]^−^, 179.0350 [M−H–C_6_H_10_O_5_–C_9_H_6_O_2_–H_2_O–C_6_H_6_O_2_]^−^, 135.0446 [M−H–C_6_H_10_O_5_–C_9_H_6_O_2_–H_2_O–C_6_H_6_O_2_–CO_2_]^−^
21	20.2	13-*O*-Coumaroylplumieride	C_30_H_32_O_14_	616.1792	617.1863 (617.1865)	455.1332 [M + H–C_6_H_10_O_5_]^+^, 437.1233 [M + H–C_6_H_10_O_5_–H_2_O]^+^, 309.0960 [M + H–C_6_H_10_O_5_–H_2_O–C_9_H_4_O]^+^, 291.0858 [M + H–C_6_H_10_O_5_–2H_2_O–C_9_H_4_O]^+^, 231.0643 [M + H–C_6_H_10_O_5_–2H_2_O–C_9_H_4_O–C_2_H_4_O_2_]^+^, 147.0430 [M + H–C_6_H_10_O_5_–2H_2_O–C_9_H_4_O–C_2_H_4_O_2_–C_4_H_4_O_2_]^+^	615.1725 (615.1719)	445.1145 [M−H–C_8_H_10_O_4_]^−^, 163.0410 [C_9_H_8_O_3_–H]^−^
22	21.6	Trihydroxy-octadecadienoic acid	C_18_H_32_O_5_	328.2250	–	–	327.2181 (327.2177)	–

*Theoretical accurate mass

#### In vitro* anti-inflammatory activity*

### Inhibition of LPS-induced NO release in RAW264.7 macrophages

**Fig. 2:**
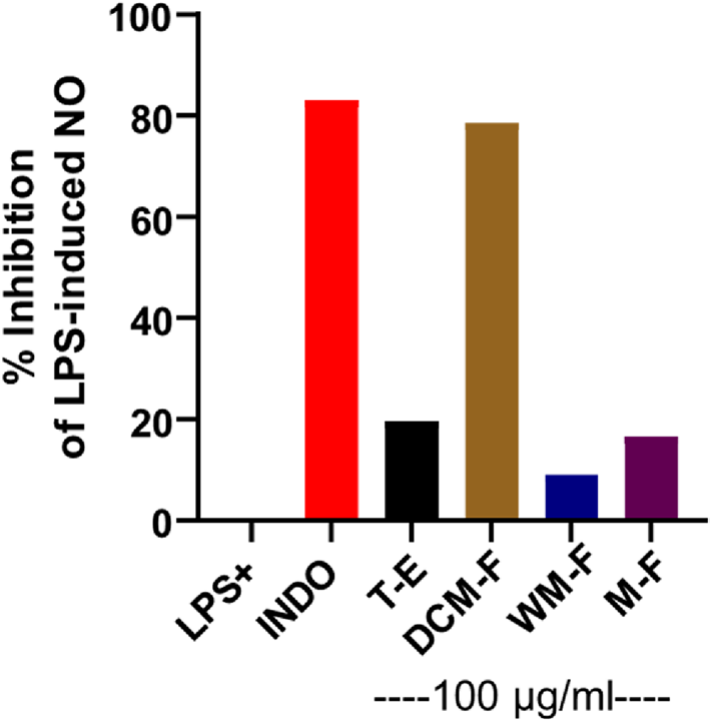
Preliminary screening of NO inhibition in RAW 264.7 macrophages by total extracts and fractions (100 µg/mL) from *Plumeria obtusa* L aerial parts*.* Cells were treated as indicated with 100 ng/ml LPS in the presence of extracts (T-E, DCM-F, WM-F or M-F) or 0.25 mM indomethacin (INDO) for 24 h and then % NO inhibition was determined by Griess assay as described in the Materials and Methods section

As the aim of the present study was to evaluate the anti-inflammatory potency of *P. obtusa* L. extract and fractions, we commenced this evaluation by preliminary screening for NO inhibition activity using a single high dose of each extract/fraction (Fig. 2).

**Fig. 3:**
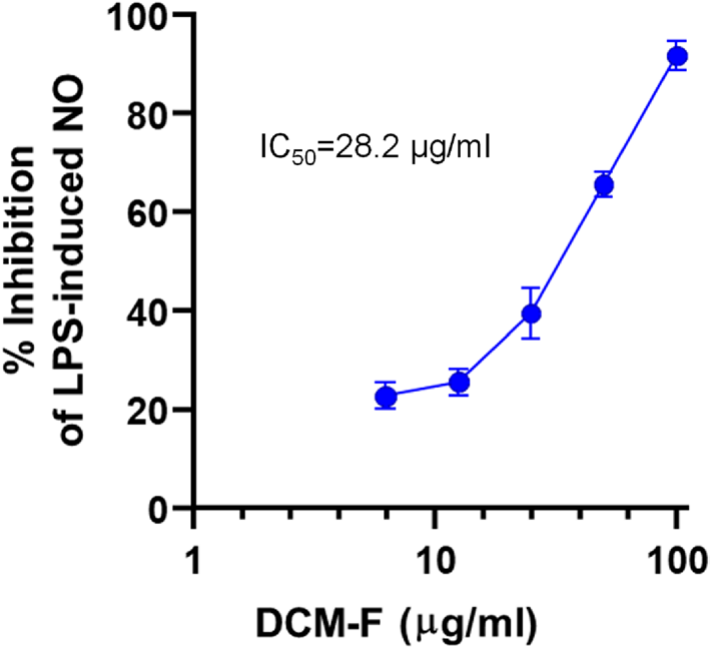
Concentration response curve for estimating IC_50_ of NO inhibition in RAW 264.7 macrophages by DCM-F aerial parts. Cells were cultured and treated as detailed in the materials and methods section. IC_50_ was estimated using the concentration response curve using GraphPad Prism as described in the Materials and Methods section

As revealed from Griess assay of the preliminary screening, DCM-F was the most active suppressor of LPS-induced NO release in RAW 264.7 macrophages among other tested samples (Fig. 2). Therefore, this fraction was subjected to further testing of concentration-dependent effects to determine its IC_50_ of NO inhibition. Following statistical analysis on GraphPad Prism using non-linear regression curve fit, IC_50_ was found to be 28.2 µg/mL (Fig. 3).

### iNOS western blotting in RAW 264.7 treated with LPS in the presence of DCM-F

**Fig. 4:**
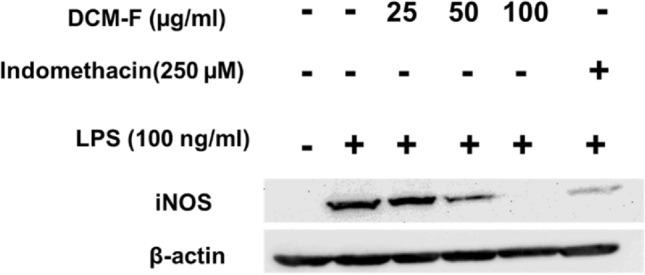
Protein expression analysis of iNOS by Western blotting of cell lysates from RAW 264.7 treated with DCM-F in the presence of LPS. Cells were cultured and treated as described in the Materials and Methods section

The inhibition of NO by DCM-F was further elucidated using Western blotting of cell lysates of RAW264.7 macrophages. As shown in Fig. 4, the LPS-induced upregulation of iNOS protein expression was inhibited after treatment with increasing concentrations of DCM-F. As shown in the blot, concentration dependent inhibition of iNOS was achieved and completely inhibited at 100 µg/mL.

### In vivo* effect of DCM-F on LPS-induced acute lung injury in mice*

The effect of DCM-F (100 mg mg/kg b.wt) and (200 mg/kg b.wt) on the levels of catalase and MDA was investigated. The results revealed that treatment of the animals with DCM-F (200 mg/kg) successfully reduced the levels of MDA to normal values compared to LPS group and increased the level of catalase enzyme in lung tissues to promising values as shown in Table 2.

**Table 2:** Effect of DCM-F (100 mg/kg b.wt) and (200 mg/kg b.wt) on the levels of catalase and MDA in LPS-ALI in mouse lungs

Groups	MDA (mmol/mg protein)	Catalase (U/mg protein)
Control negative	51.40 ± 5.67	277.20 ± 11.93
LPS	172.80 ± 5.76^a^	106.97 ± 3.48^a^
LPS + DEXA (2 mg/kg)	70.83 ± 9.03^ab^	228.57 ± 6.82^ab^
LPS + DCM-F (100 mg/kg)	80.00 ± 5.90^ab^	229.07 ± 4.48^ab^
LPS + DCM-F (200 mg/kg)	53.67 ± 7.87^b^	278.53 ± 6.14^b^

*ALI* acute lung injury, *LPS* lipopolysaccharide, *DEXA* dexamethasone, *DCM-F* dichloromethane fraction, *MDA* malondialdehyde. Results are expressed as Mean ± SD. Statistical analysis was carried out by one-way ANOVA followed by Duncan’s multiple comparison test

^a^Significant difference from normal control group at *P* < 0.05

^b^Significant difference from LPS group at *P* < 0.05

^ab^Significant difference from normal control group and LPS group at *P* < 0.05

### Effects on the levels of cytokines

The animals treated with DCM-F (200 mg/kg) exhibited a significant reduction in the proinflammatory cytokines including TNF-α and IL-6 as shown in Table 3.

**Table 3:** Effect of DCM-F (100 mg/kg b.wt) and (200 mg/kg b.wt) on the levels of TNF-α and IL-6 in LPS-ALI in mouse lungs

Groups	TNF-α (pg/mg protein)	IL-6 (pg/mg protein)
Control negative	16.80 ± 1.53	35.73 ± 1.66
LPS	82.53 ± 4.07^a^	122.67 ± 2.15^a^
LPS + DEXA (2 mg/kg)	32.63 ± 5.92^ab^	49.87 ± 4.28^ab^
LPS + DCM-F (100 mg/kg)	41.63 ± 1.47^ab^	54.63 ± 1.77^ab^
LPS + DCM-F (200 mg/kg)	20.33 ± 4.62^b^	39.13 ± 1.96^b^

*ALI* acute lung injury, *LPS* lipopolysaccharide, *DEXA* dexamethasone, *DCM-F* dichloromethane fraction, *TNF-α* tumor necrosis factor alpha, *IL-6* interleukin-6. Results are expressed as Mean ± SD. Statistical analysis was carried out by one-way ANOVA followed by Duncan’s multiple comparison test

^a^Significant difference from normal control group at *P* < 0.05

^b^Significant difference from LPS group at *P* < 0.05

^ab^Significant difference from normal control group and LPS group at *P* < 0.05

### Effects on other inflammatory mediators

The effect of DCM-F on the levels of iNOS and NO was investigated the results showed that treatment of animals with a dose of 200 mg/kg successfully reduced the levels of iNOS and NO to normal values compared to LPS group as shown in Table 4.

**Table 4:** Effect of DCM-F (100 mg/kg b.wt) and (200 mg/kg b.wt) on the levels of iNOS and NO in LPS-ALI in mouse lungs

Groups	iNOS (ng/mg protein)	NO (nmol /mg protein)
Control negative	4.97 ± 0.12	99.37 ± 5.57
LPS	19.70 ± 0.82^a^	251.63 ± 13.92^a^
LPS + DEXA (2 mg/kg)	7.19 ± 1.06^ab^	141.73 ± 7.04^ab^
LPS + DCM-F (100 mg/kg)	9.60 ± 0.85^ab^	150.00 ± 10.81^ab^
LPS + DCM-F (200 mg/kg)	5.30 ± 0.91^b^	109.07 ± 8.39^b^

*ALI* acute lung injury, *LPS* lipopolysaccharide, *DEXA* dexamethasone, *DCM-F* dichloromethane fraction, *iNOS* inducible nitric oxide synthetase, *NO* nitric oxide. Results are expressed as Mean ± SD. Statistical analysis was carried out by one-way ANOVA followed by Duncan’s multiple comparison test.

^a^Significant difference from normal control group at *P* < 0.05

^b^Significant difference from LPS group at *P* < 0.05

^ab^Significant difference from normal control group and LPS group at *P* < 0.05

### Light microscopic observations

**Fig. 5:**
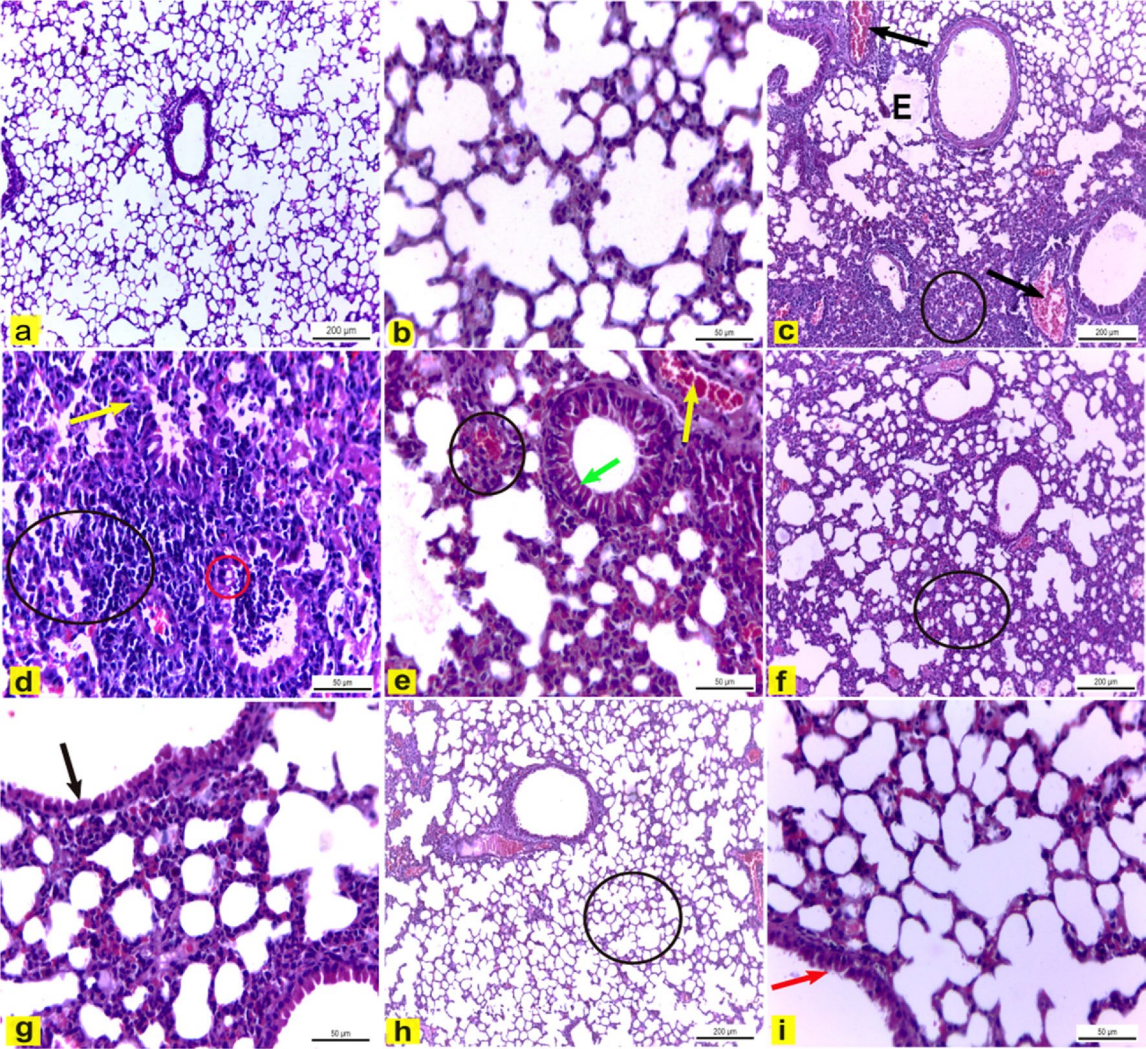
Lung tissue of mice. **a**, **b** Control mice (Group I) showing normal pulmonary architecture. H&E stain X100 and X400 respectively. **c**, **d** LPS-treated mice (Group II) revealed **(c)** Edema (E), dilated and congested blood capillaries (black arrow), and infiltration of inflammatory cells (circle). H&E stain X100. **d** Thick alveolar wall (black circle), some bronchioles had cytoplasmic vacuolation of their lining epithelium (red circle), and degenerated lining epithelium of some bronchioles was observed (yellow arrow). H&E stain X400. **e** LPS plus DEXA treated mice (Group III) showing narrowing of blood capillaries (yellow arrow), reduction of inflammatory area (circle), and restoring the normal lining epithelium of the pulmonary bronchioles (green arrow). H&E stain X400. **f**, **g** LPS plus DCM-F (100 mg) treated mice (Group IV) showing **f** reduced inflammatory cells (circle). H&E stain X100. **g** The normal lining epithelium of bronchioles (black arrow). H&E stain X400. **h**, **i** LPS plus DCM-F (200 mg) treated mice (Group V) revealing **g** no to rare inflammatory cells and nearly normal alveolar wall thickness (circle). H&E stain X100. **h** Nearly normal epithelial cells lined the bronchiole (red arrow). H&E stain X400. *LPS* lipopolysaccharide, *DEXA* dexamethasone, *DCM-F* dichloromethane fraction (Color figure online)

**Fig. 6:**
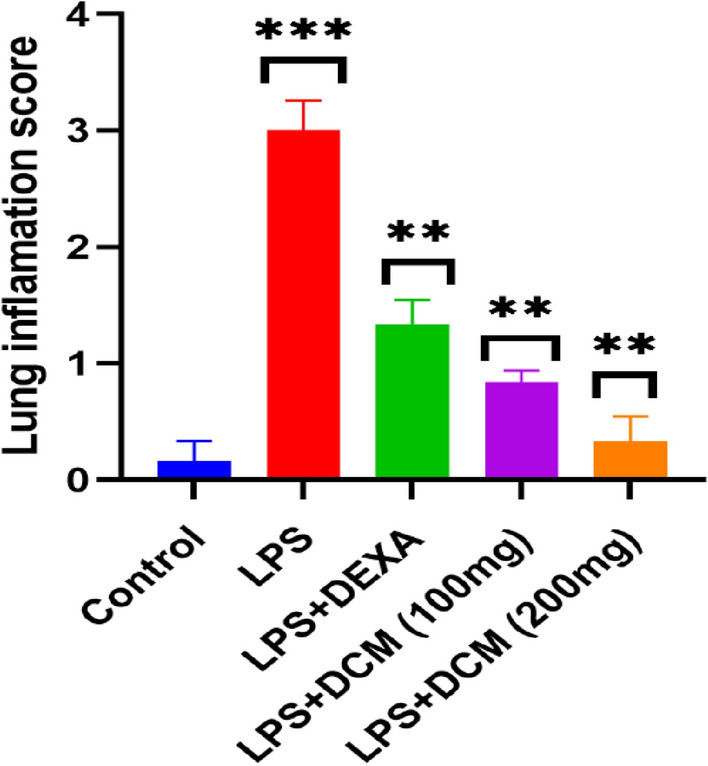
Lung inflammation scores. The results were expressed as mean ± SEM. Compared with control: ***P ˂ 0.001, compared with LPS: **P ˂ 0.01. *LPS* lipopolysaccharide, *DEXA* dexamethasone, *DCM-F* dichloromethane fraction

H&E-stained lung tissue of control mice (Group I) showed normal pulmonary architecture (Fig. 5a, b). In contrast, lung sections of LPS-treated mice (Group II) revealed several histological alterations such as lung edema, dilated blood capillaries that engorged with blood, and infiltration of inflammatory cells (Fig. 5c). Moreover, the LPS group had alveoli with thick walls, some bronchioles showed cytoplasmic vacuolation of their lining epithelium, and other bronchioles had degenerated lining epithelium (Fig. 5d). However, lung sections of mice treated with LPS plus DEXA (Group III) exhibited marked reduction of the histopathological changes which appeared as narrowing of blood capillaries, reduction of inflammatory area, and restoring the normal lining epithelium of the pulmonary bronchioles (Fig. 5e). On the other hand, mice treated with LPS plus DCM-F (100 mg/kg b.wt) (Group IV) showed ameliorated pulmonary injury with reduced inflammatory cells (Fig. 5f) and nearly normal lining epithelium of bronchioles (Fig. 5g). Finally, lung tissue of LPS plus DCM-F (200 mg/kg b.wt) treated mice (Group V) revealed lung injury recovery evidenced by nearly normal thickness of the alveolar wall, no to rare inflammatory cells (Fig. 5h), and nearly normal epithelial cells lined the bronchiole (Fig. 5i). The mean histopathological score was significantly increased in mice treated with LPS compared to the control group (*P* < 0.001). However, the histopathological score was significantly reduced by treatment of DEXA, DCM-F (100 mg/kg), and DCM-F (200 mg/kg) compared to the LPS-treated group (*P* < 0.01) (Fig. 6)

### Immunohistochemical observations

**Fig. 7:**
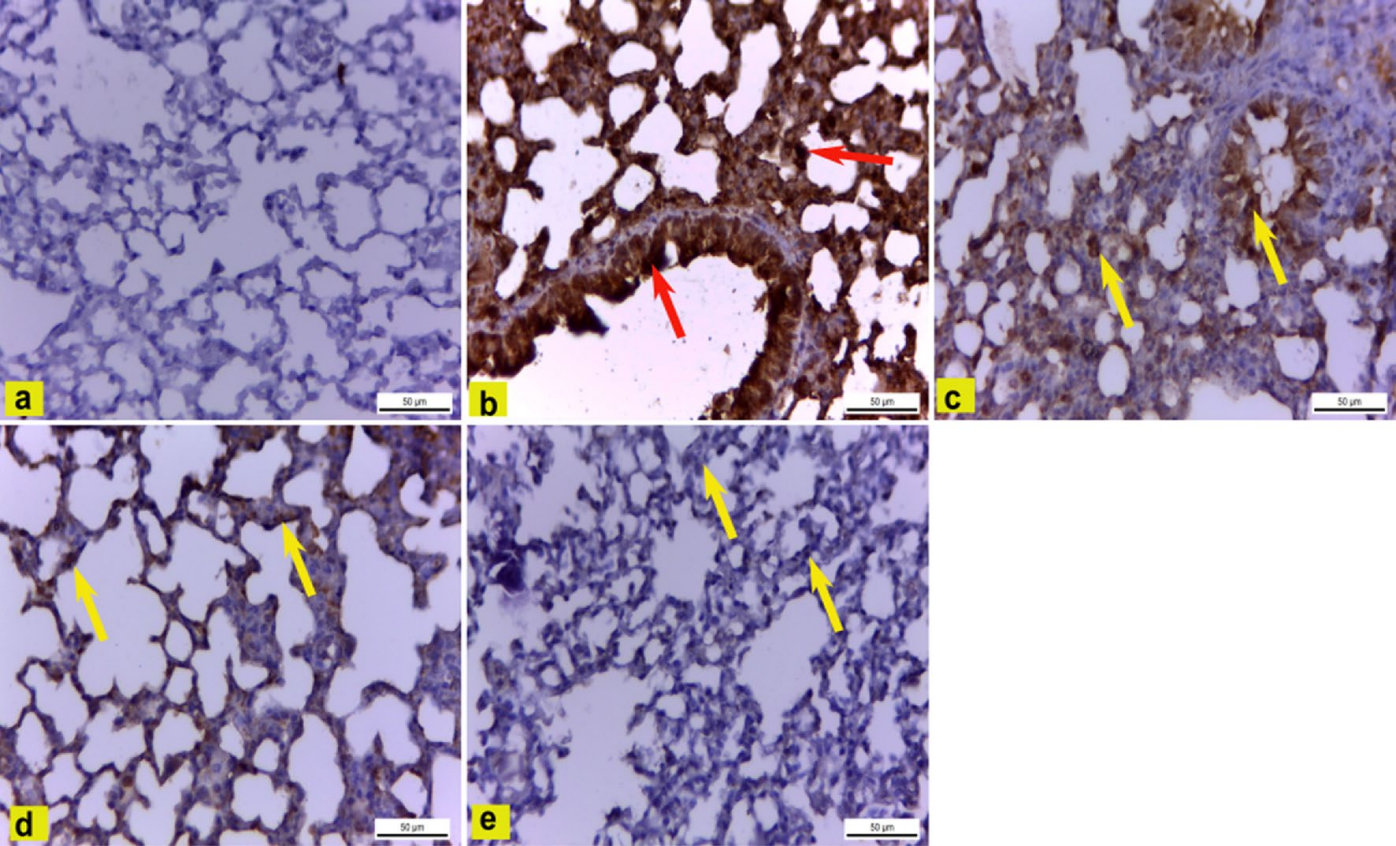
Immunohistochemically COX-2 stained pulmonary tissue (X400). **a** Control mice (Group I) showing negative COX-2 immunoreaction. **b** LPS-treated mice (Group II) revealing significant strong cytoplasmic COX-2 expression (red arrows). **c** LPS plus DEXA treated mice (Group III) exhibiting a significant decrease of COX-2 immunoreaction (yellow arrows). **d** LPS plus DCM-F (100 mg) treated mice (Group IV) showing a significant strong reduction of COX-2 expression (yellow arrows). **e** LPS plus DCM-F (200 mg) treated mice (Group V) revealing significant negligible COX-2 immune expression (yellow arrows). *LPS* lipopolysaccharide, *DEXA* dexamethasone, *DCM-F*:dichloromethane fraction (Color figure online)

**Fig.8:**
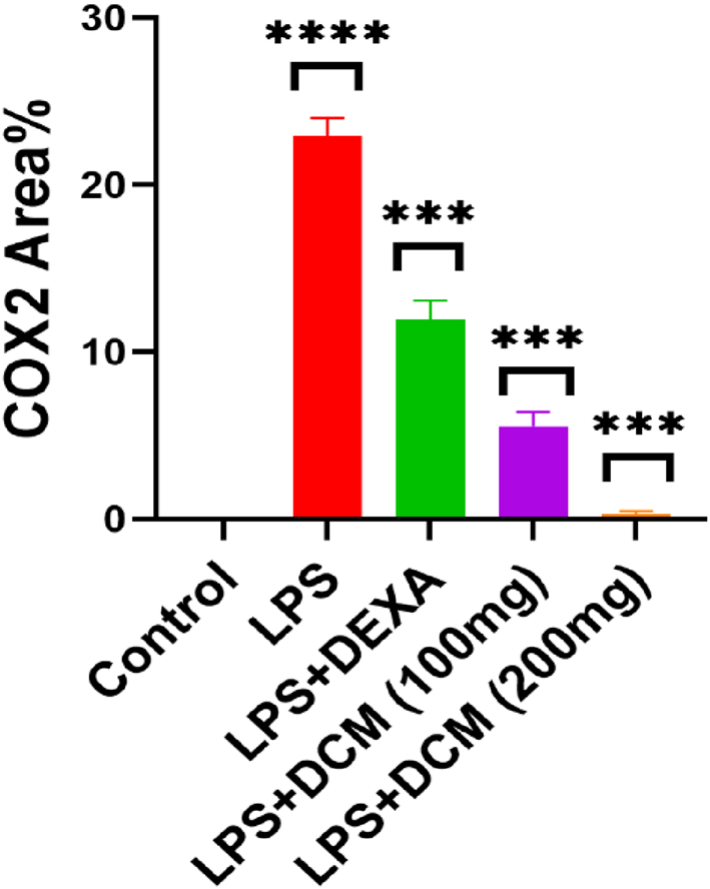
The effect of LPS, LPS plus DEXA, LPS plus DCM-F (100 mg), and LPS plus DCM-F (200 mg) on the percent area covered by COX-2-positive immunoreactive cells within pulmonary tissue of mice. Results are presented as mean ± SEM. Compared with control: ****P ˂ 0.0001, compared with LPS: ***P ˂ 0.001. LPS lipopolysaccharide, *DEXA* dexamethasone, *DCM-F* dichloromethane fraction

Immunohistochemical staining of COX-2 in lung tissue of control mice (Group I) showed negative immunoexpression (Figs. 7a, 8). Conversely, a significant strong cytoplasmic COX-2 expression in pulmonary tissue of LPS-treated mice (Group II) by 22.9 was evident, compared to the control group (Figs. 7b, 8). However, pulmonary tissue of LPS plus DEXA treated mice (Group III) revealed a significant decrease of COX-2 immunoreaction by 11.9 compared to the LPS treated group (Figs. 7c, 8). Moreover, there was a significant strong reduction of COX-2 expression in lung tissue of mice treated with LPS plus DCM-F (100 mg/kg) (Group IV) by 5.5 compared to the LPS treated group (Figs. 7d, 8). Finally, pulmonary tissue of LPS plus DCM-F (200 mg/kg) treated mice (Group V) showed significantly negligible COX-2 immuno-expression by 0.3 in comparison with the LPS treated group (Figs. 7e, 8).

### Docking studies

Both iNOS and prostaglandin E synthase-1 target receptors (1M9T and 5K0I, respectively) were analyzed using the MOE working window. This was done to investigate the crucial amino acids that bound to the co-crystallized inhibitor in each case and were responsible for producing the antagonistic activity as well.

Concerning the iNOS protein (PDB ID: 1M9T); it was found that the co-crystallized inhibitor bound Met368, Trp366, and Gly365 amino acids of the deep binding pocket at 2.98, 2.83, and 2.99 Å (all are H-bonds). The binding score of the redocked co-crystallized inhibitor was -5.73 kcal/mol (RMSD = 1.31). Notably, protoplumericin A achieved a binding score of -8.77 kcal/mol (RMSD = 1.75) and got stabilized through the formation of only one H-bond with Met368 at 2.99 Å. On the other hand, 13-*O*-coumaroylplumeride binding score was -8.69 kcal/mol (RMSD = 1.77) with the formation of two H-bonds with Trp366 and Arg193 at 2.89 and 3.43 Å, respectively. Besides, it formed a pi-H bond with Trp457 (Table 5).

**Table 5:**
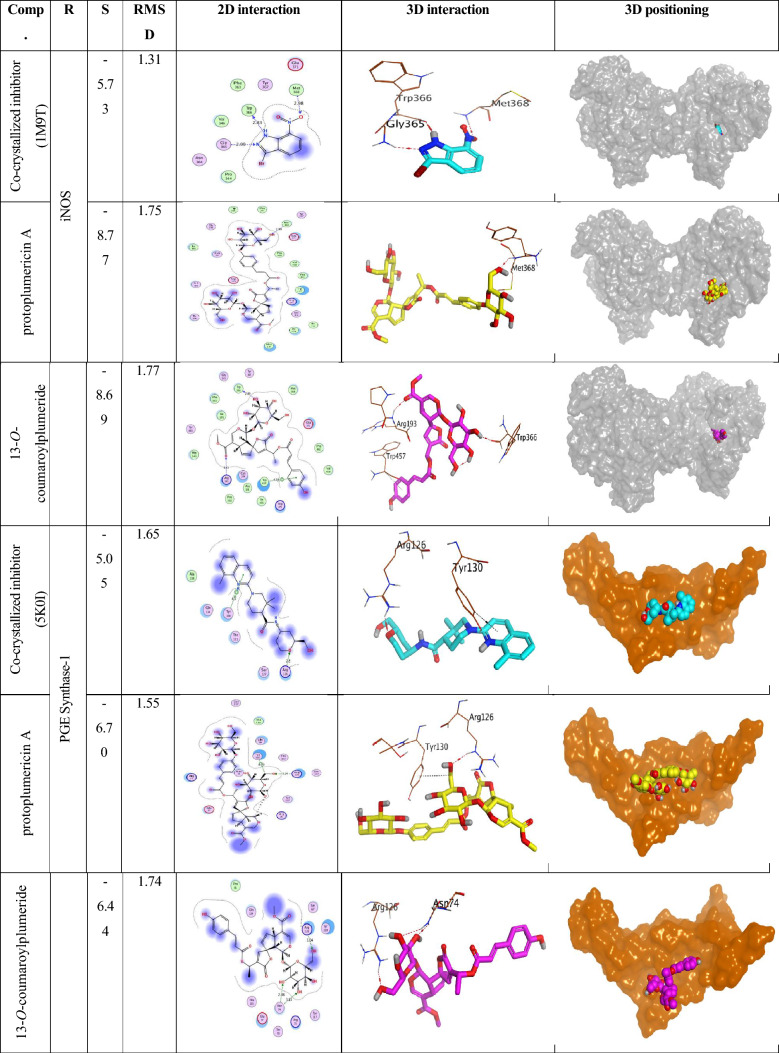
Binding scores, RMSD, 3D interactions, and 3D positioning of protoplumericin A and 13-*O*-coumaroylplumeride inside the binding pockets of the iNOS (1M9T) and prostaglandin E synthase-1 (5K0I) target receptors

*R* receptor, *S* score of a compound within the receptor binding pocket (kcal/mol)

However, regarding the prostaglandin E synthase-1 protein (PDB ID: 5K0I); it was clear that the co-crystallized inhibitor bound Arg126 and Tyr130 amino acids of the surface binding site at 2.80 (H-bond) and 4.10 (pi-H bond) Å. The binding score of the redocked co-crystallized inhibitor was -5.05 kcal/mol (RMSD = 1.65). Moreover, protoplumericin A got a binding score of -6.70 kcal/mol (RMSD = 1.55) and formed one H-bond with Arg126 at 3.24 and one H-pi bond with Tyr130 at 4.29 Å. Furthermore, 13-*O*-coumaroylplumeride showed a binding score of -6.44 kcal/mol (RMSD = 1.74) with the formation of three H-bonds with Arg126 (1) and Asn74 (2) at 3.04, 2.88, and 3.13 Å, respectively (Table 5).

## Discussion

The present study assessed the effect of *P. obtusa* L. aerial parts against LPS-induced ALI in mice through a bioactivity-guided approach. The total methanolic extract was defatted with dichloromethane to give DCM-F and the remaining was fractionated by Diaion HP20 column chromatography to 3 fractions: W-F, 50% MW-F and M-F. Among the tested fractions, DCM-F was the most active suppressor of LPS-induced NO release in RAW 264.7 macrophages. Accordingly, DCM-F was tested for its ability to mitigate the LPS-induced ALI in mice.

To investigate the different mechanisms and pathways of ALI, various experimental models have been established, the most common of which is the endotoxin (bacterial LPS) model as it is very fast and simple (Jansson et al. 2005). After intranasal administration of LPS, inflammation of the lung is induced due to damage of lung parenchyma, generation of proteases and reactive oxygen and nitrogen species, activation of lung macrophages and neutrophils in the interstitial and alveolar parts of the lung. At the end, vascular injury with diffuse alveolar damage and hemorrhage, edema, and fibrin deposition occur (Puljic et al. 2007; Shokry et al. 2022).

After the entrance of LPS into the lung tissues, significant increase in the reactive oxygen species (ROS) occurs (Wu et al. 2020), which subsequently led to reduction in the level of catalase and increase in the level of MDA, and accompanied by neutrophil infiltration in the lung (Li et al. 2015). Our results showed that DCM-F administration significantly inhibited lung inflammation, increased the level of catalase and decreased that of MDA. These findings indicated that DCM-F has a potent antioxidant effect to protect against exposure to excessive amounts of ROS for a long time. It may also explain the effect of DCM-F treatment in the control of pulmonary dysfunction, tissue injury, intracellular edema, and lipid peroxidation that have been occurred after ROS exposure (Yu et al. 2015).

Catalase, one of the most effective antioxidant enzymes present in most aerobic cells, catalyzes hydrogen peroxide to water and oxygen. It is present and expressed by high amount in the bronchiolar and alveolar epithelial cells due to its antioxidant capacity in the normal lungs (Ansar et al. 2020). Treatment with DCM-F (200 mg/kg) successfully restored the level of catalase to normal values and showed better results than the standard drug DEXA.

LPS administration leads to lung inflammation that is usually accompanied by stimulation and accumulation of cytokines (such as TNF-α and IL-6) within the lung tissues (Cribbs et al. 2010). TNF-α has been involved as the first cytokine appear in many lung diseases; asthma, chronic bronchitis, chronic obstructive pulmonary disease, acute lung injury (ALI), and acute respiratory distress syndrome (Yang et al. 2012). Within the lung tissue, TNF-α is generated at high level and initiate different inflammatory processes which in turn lead to the development of inflammatory responses that are the main fingerprint of many diseases. On the other hand, IL-6 is one of the most active cytokines which play important role in the inflammatory processes. Increased IL-6 level after LPS exposure is usually occur and followed by different pathological conditions such as lung injuries leading to acute respiratory distress syndrome. LPS-induced lung injury depends mainly on cytokines infiltration into the endothelium with high permeability and exudate accumulation triggers systemic reactions, including septic shock. In this study, we found that the levels of TNF-α, and IL-6 were upgraded in tissues after LPS administration, while treatment with DCM-F (200 mg/kg) successfully decreased their levels to normal values.

Inducible nitric oxide synthases (iNOS), the enzyme responsible for production of NO, is one of the most important constituents in the inflammatory process in the lung and alveoli inflammation (Kim et al. 2014). Accumulation of high content of NO within the lung tissues induces the release of pro-inflammatory cytokines such as TNF-α, IL-1β, IL-6, NO, and PGE2 (Bae et al. 2012). However, pre-treatment with DCM-F (200 mg/kg) strongly prevented the increase of iNOS and NO-induced by LPS. This was also followed by decrease in the amount of released cytokines.

In the present investigation, pulmonary tissues of LPS-treated mice (Group II) revealed several histological alterations compared to control mice such as lung edema, and dilated blood capillaries that engorged with blood, and infiltration of inflammatory cells. These findings agreed with (Huang et al. 2019; Tian et al. 2019; Shen et al. 2020; Liu et al. 2022; Shen et al. 2020; Huang et al. 2019; Tian et al. 2019; Wang et al. 2022) demonstrated that ALI is accompanied by many inflammatory cells’ infiltration. Stimulation of these inflammatory cells leads to the release of inflammatory mediators that interrupt pulmonary endothelial cells and pulmonary epithelial integrity leading to lung edema. Edema in lung tissues is a common indication of inflammation in both local and systemic inflammation (Giebelen et al. 2007). Furthermore, the LPS-treated group had a thick alveolar wall and degenerated lining epithelium of some bronchioles. These observations correlated to (Fan et al. 2018; Fan et al. 2018) who reported thickening of alveolar walls, and disruption of endothelial and epithelial integrity after intratracheally stimulated mice with LPS.

On the other hand, the LPS group co-treated with DEXA (Group III) revealed a marked reduction of histopathological changes compared to LPS-treated mice in form of narrowing of blood capillaries, reduction of inflammatory area, and restoration of the normal lining epithelium of the pulmonary bronchioles. These results come in line with the findings of (Shen et al. 2020; Shen et al. 2020) who reported that DEXA administration attenuated the LPS-induced lung inflammation. Our results are supported by (Saklatvala 2002 and (Rhen and Cidlowski 2005; Rhen and Cidlowski 2005) as they stated that DEXA is a powerful anti-inflammatory drug commonly used to treat various inflammatory diseases, including asthma, and chronic obstructive pulmonary disease, and acute respiratory distress syndrome.

Moreover, lung tissue of mice treated with LPS plus DCM-F (100 mg/kg) (Group IV) revealed significant prevention of acute lung inflammation and pulmonary tissue of mice treated with LPS plus DCM-F (200 mg/kg) (Group V) appeared nearly normal with significant amelioration of acute lung inflammation. These findings indicate that DCM-F has potent anti-inflammatory properties, supported by (Lotankar et al. 2016; Lotankar et al. 2016) who reported that the ethanolic extract of *P. obtusa* bark (at doses of 100, 200, and 400 mg/kg) significantly decreased the paw volume in carrageenan-induced paw edema.

The immunohistochemical examination of LPS-treated mice (Group II) showed significant cytoplasmic expression of COX-2 in pulmonary tissue compared to control mice. These results agreed with (Huang et al. 2019; Huang et al. 2019) who mentioned that COX-2 is expressed massively in the nucleus and the cytoplasm of the LPS-treated group. On the contrary, COX-2 immunoreaction was significantly reduced in mice treated with LPS plus DEXA compared to LPS-treated mice, as reported by (Huang et al. 2019; Huang et al. 2019). However, there was a significant reduction of COX-2 expression in lung tissue of mice treated with LPS plus DCM-F (100 mg/kg) (Group IV) and significant negligible COX-2 immuno-expression in mice treated with LPS plus DCM-F (200 mg/kg) (Group V) compared to LPS-treated mice.

*P. obtusa* was traditionally used to treat ulcers, wounds and skin diseases, and a broad spectrum of biological activities have been reported (Bihani 2021). Among 22 compounds identified by LC-DAD-QToF, DCM-F was found to contain iridoids, phenolic acids, and flavonoids as major constituents. Based on the molecular docking, it was clear that the iridoid derivatives; protoplumericin A and 13-*O*-coumaroylplumeride achieved superior binding scores compared to the co-crystallized inhibitor of both iNOS and prostaglandin E synthase-1 target receptors. Besides, they bound the same crucial amino acids of the binding pockets of both receptors indicating the greatly expected antagonistic activities as well.

## Conclusion

The present study revealed that DCM-F is a potential agent to attenuate the LPS-induced ALI, and its effect may be attributed, at least in part, to its antioxidant and anti-inflammatory effects. The DCM-F inhibited the secretion of proinflammatory cytokines; IL-6 and TNF-α. It significantly inhibited the LPS-induced levels of the iNOS protein in the Raw264.7 macrophage cell line. In depth study of the mechanism(s) of action of DCM-F will be reported in the forthcoming article.

## Data Availability

Data supporting findings are presented within the manuscript. Enquiries about data availability should be directed to the authors.
