# Editorial Expression of Concern: Bioactive fraction from *Plumeria obtusa* L. attenuates LPS‑induced acute lung injury in mice and inflammation in RAW 264.7 macrophages: LC/QToF‑MS and molecular docking

**DOI:** 10.1007/s10787-025-02058-5

**Published:** 2025-12-03

**Authors:** Yousra T. Eloutify, Riham A. El‑Shiekh, Khaled Meselhy Ibrahim, Ahmed R. Hamed, Ahmed A. Al‑Karmalawy, Aya A. Shokry, Yasmine H. Ahmed, Bharathi Avula, Kumar Katragunta, Ikhlas A. Khan, Meselhy R. Meselhy

**Affiliations:** 1 Department of Pharmacognosy, Faculty of Pharmacy, Cairo University, Kasr El Aini St., Cairo, 11562 Egypt; 2 Chemistry of Medicinal Plants Department and Biology Unit, Central Lab for the Pharmaceutical and Drug Industries Research Institute, National Research Centre, 33 El‑Bohouth St, Dokki, 12622 Egypt; 3 Pharmaceutical Chemistry Department, Faculty of Pharmacy, Ahram Canadian University, 6th of October City, Giza, 12566 Egypt; 4 Department of Pharmacology, Faculty of Veterinary Medicine, Cairo University, Cairo, Egypt; 5 Cytology and Histology Department, Faculty of Veterinary Medicine, Cairo University, Giza, Egypt; 6 National Center for Natural Products Research, School of Pharmacy, University of Mississippi, University, MS 38677 USA; 7 Division of Pharmacognosy, Department of BioMolecular Sciences, School of Pharmacy, University of Mississippi, University, MS 38677 USA

**Editorial Expression of Concern: Inflammopharmacology (2023) 31:859–875** 10.1007/s10787-023-01144-w

The Editors-in-Chief would like to alert readers that there are some concerns related to this article. Concern has been raised regarding some of the images in this article overlap with images in another article [1]. These are Fig. 7a of this article, which overlaps with Fig. 6a [1], and Fig. 7b of this article, which overlaps with Fig. 6g [1. The authors provided the corrected images, however the journal was unable to validate these. Readers are advised to interpret the details of this article with caution.

Riham A. El-Shiekh, Khaled Meselhy Ibrahim, Ahmed R. Hamed, Yasmine H. Ahmed and Meselhy R. Meselhy all agreed with this editorial expression of concern. Aya A. Shokry did not disagree with this editorial expression of concern but did not fully agree with the wording. We did not receive responses from Ahmed A. Al-Karmalawy, Bharathi Avula, Kumar Katragunta and Ikhlas A. Khan. We were unable to get in touch with Yousra T. Eloutify.

1. El-Shiekh, R.A., Nabil, G., Shokry, A.A. et al. Arabincoside B isolated from Caralluma arabica as a potential anti-pneumonitis in LPS mice model. Inflammopharmacol 31, 1437–1447 (2023).

